# The Diagnostic Performance of Transvaginal Ultrasound for Posterior Compartment Endometriosis Compared to Laparoscopic and Histopathological Findings: A Systematic Review

**DOI:** 10.3390/healthcare13202548

**Published:** 2025-10-10

**Authors:** Roxana-Denisa Capraș, Iulia Clara Badea, Mădălina Moldovan, Adriana Ioana Gaia-Oltean, Alexandru-Florin Badea, Teodora Telecan

**Affiliations:** 1 Department of Anatomy and Embryology, “Iuliu Hațieganu” University of Medicine and Pharmacy, 400012 Cluj-Napoca, Romania; capras.roxana@umfcluj.ro (R.-D.C.); moldovan.madalina@elearn.umfcluj.ro (M.M.); afbadea84@gmail.com (A.-F.B.); t.telecan@gmail.com (T.T.); 2 Department of Prevention in Dental Medicine, Faculty of Dental Medicine, “Iuliu Hatieganu” University of Medicine and Pharmacy, 400012 Cluj-Napoca, Romania; 3 Department of Obstetrics and Gynecology, University of Medicine and Pharmacy Iuliu Hatieganu, 400347 Cluj-Napoca, Romania; adriana.gaia@umfcluj.ro; 4 Department of Obstetrics and Gynecology, “Regina Maria” Hospital, 400609 Cluj-Napoca, Romania; 5 Department of Pathology, County Emergency Clinical Hospital, 400347 Cluj-Napoca, Romania

**Keywords:** deep infiltrating endometriosis, transvaginal ultrasound, diagnostic accuracy, posterior compartment, rectosigmoid endometriosis, uterosacral ligaments, pouch of Douglas obliteration

## Abstract

**Background:** Deep infiltrating endometriosis (DIE) frequently affects the posterior pelvic compartment, where accurate non-invasive imaging is essential for diagnosis and surgical planning. **Aim:** This systematic review evaluates the diagnostic performance of transvaginal ultrasound (TVUS) in detecting posterior compartment DIE, specifically rectosigmoid lesions, uterosacral ligament involvement, and pouch of Douglas obliteration. **Material and Methods:** A comprehensive literature search of PubMed, Scopus, and Web of Science was performed for studies published between 2015 and 2025. Eligible studies assessed the accuracy of TVUS for posterior compartment DIE using laparoscopy and histology as reference standards. Data on sensitivity, specificity, and overall diagnostic accuracy were extracted or derived. The study’s quality was evaluated using the QUADAS-2 tool. **Results:** Thirty eligible studies were included. The mean sensitivities and specificities reported in the included studies reached 83.05% and 90.53% for rectosigmoid disease, 78.07% and 90.49% for uterosacral ligament involvement, and 79.58% and 89.75% for pouch of Douglas obliteration, respectively. Adjunctive techniques such as gel sonovaginography, rectal water contrast, or saline instillation into the pouch of Douglas were described, but their use was inconsistent. Marked heterogeneity in patient preparation, scanning protocols, and reporting limited comparability across studies. Despite this, TVUS demonstrated diagnostic performance within a similar range to that reported for MRI in prior systematic reviews, with the advantages of lower cost, accessibility, and integration into routine gynecological practice. **Conclusions:** TVUS is consistently reported as a reliable and cost-effective imaging modality and, in line with international guidelines, should be considered the first-line option for posterior compartment DIE, though further standardization of scanning and reporting protocols is needed to optimize reproducibility and clinical utility.

## 1. Introduction

Endometriosis is a chronic, estrogen-dependent inflammatory condition affecting approximately 10% of women of reproductive age worldwide [1]. The prevalence is notably higher in specific subgroups, with endometriosis being identified in 28–42% of women presenting with chronic pelvic pain and in 24–31% of those experiencing infertility [2]. Beyond its clinical implications, endometriosis imposes a substantial socioeconomic burden. In the United States, direct medical costs per patient per year typically range from USD 13,000 to USD 20,000, while indirect costs—primarily attributed to absenteeism and reduced workplace productivity—can be equally impactful, being estimated between USD 4500 and USD 15,000 annually per patient [3,4].

Deep infiltrating endometriosis (DIE) is characterized by endometriotic lesions that penetrate more than 5 mm beneath the peritoneal surface, frequently involving the muscularis propria of pelvic organs or retroperitoneal structures. These lesions are often nodular and fibrotic in nature, contributing to severe pain symptoms and potential impairment of organ function and fertility [5]. Among the most commonly affected extraovarian sites are those of the posterior pelvic compartment, with cul-de-sac (pouch of Douglas) obliteration (PDO) reported in approximately 37% of cases, uterosacral ligament (USL) involvement in 24%, and bowel infiltration in 23%, of which 72–90% of cases affect the rectosigmoid colon [6,7].

The current gold standard for diagnosing endometriosis is laparoscopic visualization of lesions, followed by histopathological confirmation through targeted biopsy [8]. This invasive approach allows for direct inspection of the pelvic and abdominal cavities, enabling accurate identification and classification of endometriotic implants. However, several international guidelines—including those issued by the European Society of Human Reproduction and Embryology (ESHRE), the Society of Obstetricians and Gynecologists of Canada (SOGC), and the National Institute for Health and Care Excellence (NICE)—increasingly support a less invasive diagnostic paradigm. In terms of imaging, transvaginal ultrasound (TVUS) is considered the gold-standard non-invasive technique for evaluating suspected endometriosis, particularly when it incorporates dynamic assessments such as ovarian mobility, site-specific tenderness, and the uterine sliding sign, which significantly enhance the detection of deep infiltrating endometriosis and posterior compartment involvement [9].

Given the expanding role of transvaginal ultrasound in the diagnostic work-up of endometriosis, there is a growing need to consolidate its reported performance across various anatomical sites. While numerous studies have evaluated its accuracy for detecting deep infiltrating endometriosis, particularly in the posterior compartment, the literature remains fragmented, with considerable variability in methodology, patient selection, and technical execution. Consequently, the reported diagnostic performance of transvaginal ultrasound varies, with sensitivities ranging from 76% to 94%, specificities from 94% to 99%, and overall accuracy averaging around 86% [10,11].

In this context, the present review aims to provide a comprehensive and up-to-date synthesis of the diagnostic performance of TVUS in identifying cul-de-sac obliteration, uterosacral ligament involvement, and rectosigmoid lesions—highlighting key sonographic markers, procedural enhancements, and heterogeneity in clinical practice.

## 2. Materials and Methods

This review was conducted in accordance with the PRISMA 2020 guidelines (Supplementary Table S1). For clarity, the review question was structured using the PIRT (Participants, Index test, Reference standard, Target condition) framework: Participants were women with suspected deep infiltrating endometriosis of the posterior compartment; the Index test was transvaginal ultrasound (TVUS); the Reference standard was laparoscopic and/or histopathological confirmation; and the Target condition was deep infiltrating endometriosis involving the rectosigmoid colon, uterosacral ligaments, or pouch of Douglas. Diagnostic performance outcomes of interest were sensitivity, specificity, and accuracy, as reported by the included studies. The review protocol was not prospectively registered. Two reviewers (T.T. and R.-D.C.) independently screened the titles and abstracts of all retrieved records and subsequently assessed the full-text articles for eligibility. Discrepancies were resolved through discussion, with arbitration by a third reviewer when necessary. No automation tools were employed in the selection process. A comprehensive search of PubMed, Scopus, and Web of Science (WoS) was performed to identify studies evaluating the diagnostic accuracy of transvaginal ultrasound for posterior compartment endometriosis. The search was completed in August 2025. The PubMed strategy combined both MeSH terms and free-text keywords: (“Transvaginal Ultrasonography” [Mesh] OR “transvaginal ultrasound” OR “TVUS”) AND (“Endometriosis” [Mesh] OR “deep infiltrating endometriosis” OR “DIE”). The Scopus query was as follows: TITLE-ABS-KEY (“transvaginal ultrasound” OR “transvaginal ultrasonography” OR “TVUS”) AND TITLE-ABS-KEY (“deep infiltrating endometriosis” OR “DIE” OR “endometriosis”). The Web of Science query was as follows: TS = (“transvaginal ultrasound” OR “transvaginal ultrasonography” OR “TVUS”) AND TS = (“deep infiltrating endometriosis” OR “DIE” OR “endometriosis”). The searches retrieved 1297 records in PubMed, 1318 in Scopus, and 1103 in Web of Science. We only included studies from the past decade to ensure that diagnostic accuracy estimates reflect contemporary ultrasound technology, standardized protocols, and current clinical practice. To further enhance the thoroughness of the search strategy, the reference lists of all included studies were screened manually.

Studies were eligible for inclusion if they evaluated the diagnostic accuracy of transvaginal ultrasound for detecting posterior compartment endometriosis—specifically involving the rectosigmoid, uterosacral ligaments, or pouch of Douglas obliteration—using laparoscopic and histological findings as the reference standard. In instances where diagnostic accuracy was not explicitly reported, it was derived by the review authors as the sum of true positives and true negatives divided by the total sample size. True positive and true negative counts were reconstructed from the sensitivity, specificity, and disease prevalence (or absolute case numbers) reported in the original studies. Only original studies published in peer-reviewed journals with the full-text available in English were considered for inclusion.

As such, the exclusion criteria were as follows:

- Full-text available in languages other than English.
- Designed as a systematic review, meta-analysis, comment, letter to the editor, or conference abstract.
- Published before 2015.
- Conducted on animal or experimental models.
- Protocols focused on imaging techniques other than transvaginal ultrasound.
- Solely targeted treatment options or fertility assessment.
- Did not evaluate endometriosis of the posterior pelvic compartment.

During the eligibility assessment, we excluded studies that focused primarily on radiomics or artificial intelligence, those designed as questionnaires or interobserver variability assessments, and those that did not report on the diagnostic accuracy of transvaginal ultrasound nor data from which the diagnostic accuracy could be later derived. We also excluded studies centered on lesion mapping without accuracy evaluation and those comparing accuracy exclusively with other imaging modalities rather than with laparoscopic and histological findings.

**Figure 1:**
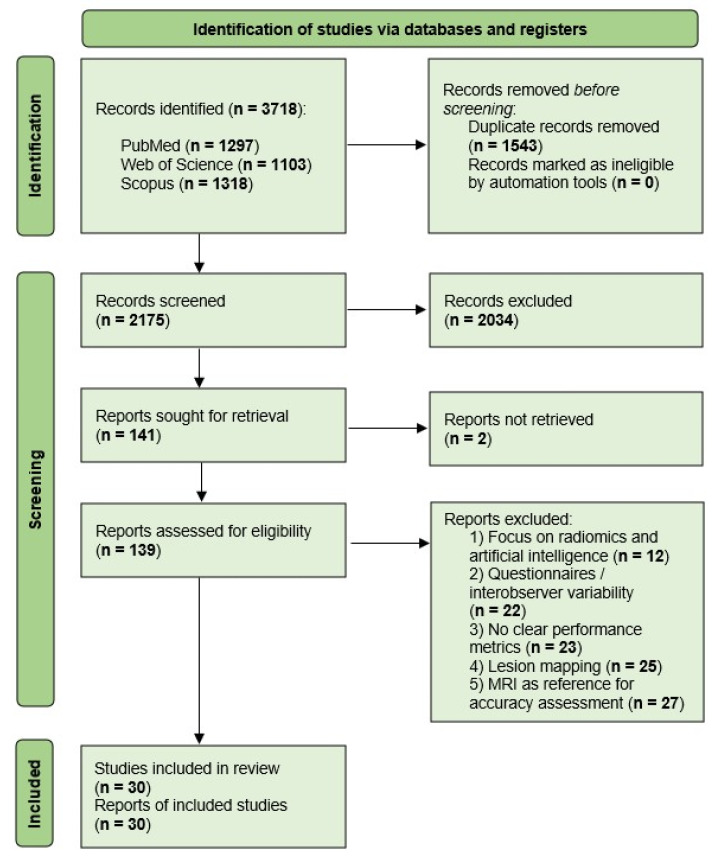
PRISMA flow diagram of study selection process. Following screening and full-text review, 30 studies were included in this systematic review. In this review, each study corresponds to a single report; therefore, the number of included studies (n = 30) is equal to the number of reports (n = 30).

The search process was synthetized in a Preferred Reporting Items for Systematic Reviews and Meta-Analyses (PRISMA)-type flowchart (Figure 1).

For each included study, diagnostic performance was assessed according to sensitivity, specificity, and accuracy, which were extracted from each study whenever available. When technical or demographic parameters (e.g., probe frequency, bowel preparation, or mean age) were not specified, these were documented as “not reported.”

In addition, study-level characteristics were collected, including first author, year of publication, country, study design (prospective/retrospective), sample size, reference standard, anatomical site assessed (rectosigmoid, uterosacral ligaments, pouch of Douglas), ultrasound probe characteristics, and use of adjunctive techniques (e.g., gel sonovaginography, rectal water contrast, saline infusion). When information was missing or unclear, data were noted as not reported, and no assumptions were made beyond what was available in the published report.

To assess the methodological quality and risk of bias of the included studies, we applied the Quality Assessment of Diagnostic Accuracy Studies-2 (QUADAS-2) tool [12]. Two reviewers independently performed the assessment, with discrepancies resolved through discussion and, if necessary, consultation with the senior author.

Studies were grouped according to the anatomical site assessed, specifically the rectosigmoid colon, uterosacral ligaments, and pouch of Douglas. Given the marked heterogeneity in study methodologies, patient preparation, and outcome definitions, a quantitative meta-analysis was not undertaken. Instead, the results were synthesized narratively, with the findings presented separately for each anatomical site. Potential sources of heterogeneity, including variability in bowel preparation, adjunctive imaging techniques, and criteria for defining pouch of Douglas obliteration, are explored qualitatively in the Discussion Section. No sensitivity analyses were performed, as a formal meta-analysis was not conducted.

## 3. Results

### 3.1. Study Selection and Characteristics

The initial search retrieved 3718 records, of which 2175 remained after the removal of 1543 duplicates. Of the 2175 results, 2034 were excluded during title and abstract screening due to irrelevance (imaging modality, pelvic compartment), non-English language, or publication date. This process left 141 articles for inclusion, of which 2 were not accessible in full text, thus leaving 139 articles for full-text eligibility assessment. Of these, 109 studies were excluded: 12 focused on radiomics or artificial intelligence-driven diagnostics, 23 did not explicitly report the diagnostic accuracy of transvaginal ultrasound or data from which the diagnostic accuracy could be derived, 25 centered on lesion mapping prior to surgery, 27 compared transvaginal ultrasound accuracy only with other imaging techniques, and 22 focused on questionnaires or assessing interobserver variability, thus leaving 30 studies for the final analysis. In the present review, each included study is represented by a single full-text report; therefore, the number of included studies (n = 30) is identical to the number of reports (n = 30).

Of the 30 studies included, 18 evaluated rectosigmoid endometriosis, 17 assessed uterosacral ligament involvement, and 13 investigated pouch of Douglas obliteration. Several studies addressed more than one anatomical site; therefore, the sum of studies per site exceeds the total number of studies included. The characteristics and main findings of all included studies are summarized in Table 1. Several studies did not report key technical parameters, most frequently probe frequency (n = 4) and patient characteristics such as mean age (n = 3). In addition, bowel preparation protocols were inconsistently described, with many studies omitting such details altogether. These gaps in reporting limited the ability to assess the influence of technical and preparatory factors on diagnostic performance.

**Figure 2:**
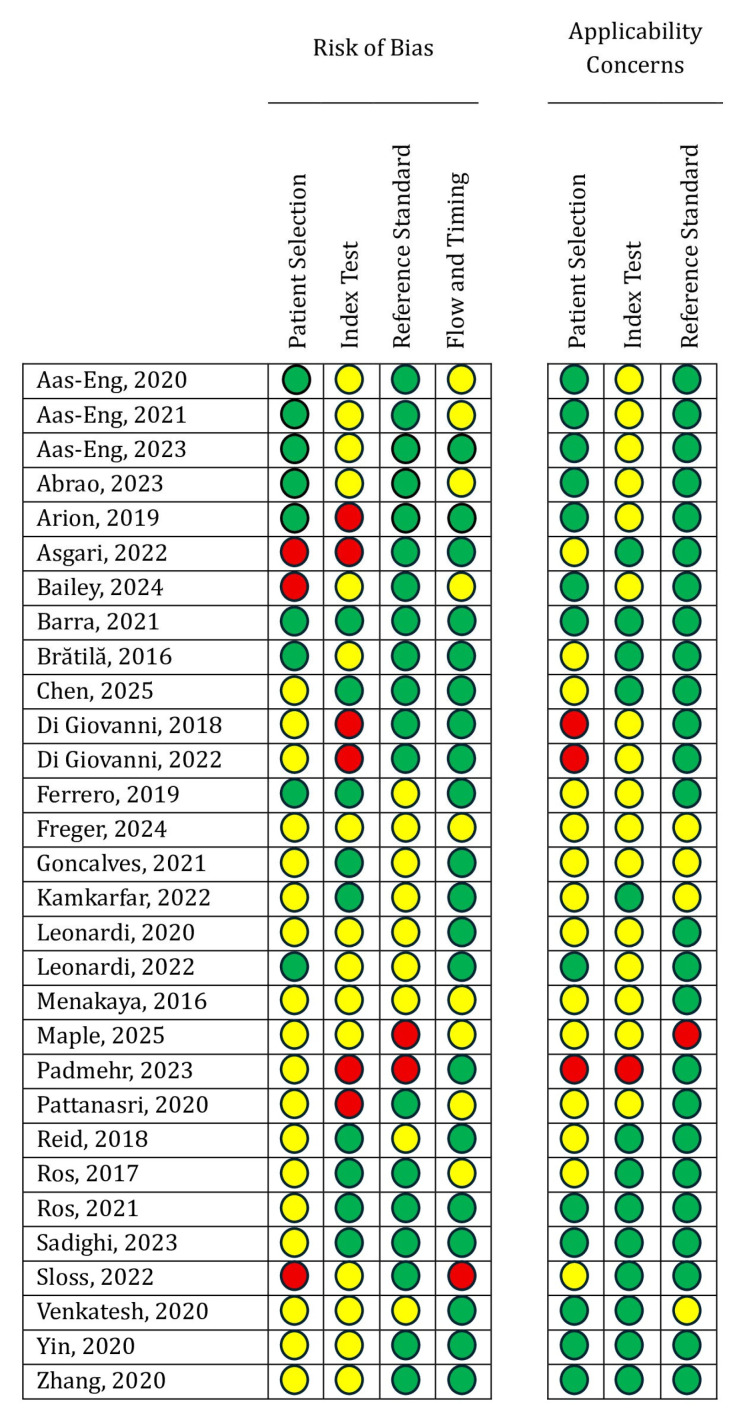
Detailed QUADAS-2 assessment of methodological quality for each included study evaluating the diagnostic accuracy of transvaginal ultrasound in posterior compartment deep infiltrating endometriosis (Green = Low bias risk; Yellow = Unclear bias risk; Red = High bias risk) [11,13,14,15,16,17,18,19,20,21,22,23,24,25,26,27,28,29,30,31,32,33,34,35,36,37,38,39,40,41].

**Figure 3:**
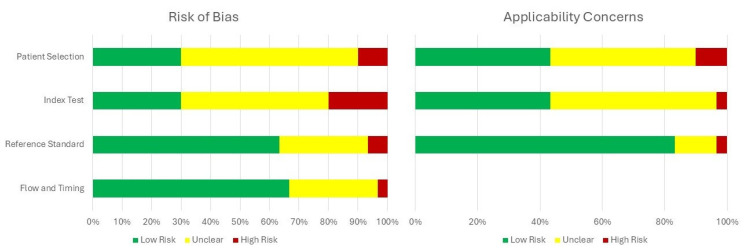
Summary of QUADAS-2 assessments across all included studies.

The risk of bias assessment using the QUADAS-2 tool is presented in Figure 2 (per-study evaluation) and Figure 3 (summary of domains). Detailed per-study justifications are provided in Supplementary Table S2. Most studies were rated as low risk for the reference standard domain, while concerns regarding patient selection and index test applicability were more frequent.

### 3.2. Diagnostic Performance of TVUS for Rectosigmoid Endometriosis

**Figure 4:**
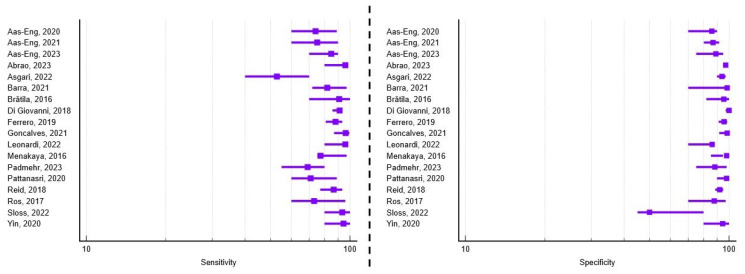
Forest plots of study-level diagnostic performance of transvaginal ultrasound for rectosigmoid deep infiltrating endometriosis. Sensitivity (**left**) and specificity (**right**) are shown with squares representing point estimates and horizontal lines denoting 95% confidence intervals [11,12,13,14,15,16,18,20,21,23,25,27,30,32,33,34,35,38,40].

A total of 18 studies met the inclusion criteria and addressed the diagnostic accuracy of transvaginal ultrasound for detecting rectosigmoid deep infiltrating endometriosis prior to surgery. Of these, 44.44% (n = 8) focused exclusively on the digestive component of the posterior compartment. Regarding study design, seven papers employed a retrospective approach, ten were prospective, and one study combined both methodologies. Moreover, 61.11% (n = 11) recruited patients from a single diagnostic and treatment center. The average number of patients per study was 228, ranging from 42 to 878, and the mean age of the study population was 34.7 years. The average reported sensitivity and specificity were 83.05% (range 52.9–96.2%) and 90.53% (range 50–100%), respectively. A visual synthesis of these results is provided in the forest plots (Figure 4), which illustrate the variability in diagnostic performance across studies despite overall high values. Additional methodological details, including patient preparation, adjunctive techniques, and technical specifications of the ultrasound examinations, are summarized in Supplementary Table S3.

Several studies evaluated the performance of transvaginal ultrasound in assessing the depth of rectosigmoid wall infiltration, particularly regarding muscular and submucosal involvement. Di Giovanni et al. [23] demonstrated excellent concordance between ultrasound and histopathology for lesion size, confirming the reliability of TVUS in preoperative assessment. Similarly, Ros et al. [35], Menakaya et al. [30], and Sloss et al. [38] reported that TVUS accurately identified muscularis invasion in 85–92% of surgically confirmed cases, highlighting its value in characterizing bowel wall involvement and guiding surgical planning.

Most studies used surgical resection of the affected bowel segment as the reference standard, while only one employed diagnostic laparoscopy with confirmatory biopsy as part of a multimodal management strategy [30]. The series by Aas-Eng et al. [13,15] emphasized the importance of assessing the distance from the lesion to the anal verge, as an anastomotic height of ≤5 cm is associated with an increased risk of leakage and fistula formation in rectal surgery. Using transvaginal ultrasound, the authors demonstrated excellent agreement with intraoperative measurements, with an intraclass correlation coefficient of 0.81, confirming the reliability of TVUS in preoperative planning for low colorectal resections and in evaluating the proximity of deep endometriotic nodules to the anal sphincter.

To enhance the diagnostic yield of transvaginal ultrasound for rectosigmoid deep endometriosis, several authors evaluated adjunctive imaging techniques that improve tissue contrast and delineation. Menakaya et al. [30] demonstrated that gel sonovaginography increased the accuracy of lesion detection, particularly in patients with restricted vaginal mobility due to severe disease. Similarly, Barra et al. [20] reported that rectal water contrast improved visualization of bowel wall layers and more accurately defined the depth of infiltration, achieving a sensitivity of 95.2% compared to 82.0% for standard TVUS (*p* = 0.003). These adjuncts appear valuable when conventional TVUS yields inconclusive findings or when multifocal mural involvement is suspected, providing added benefit in surgical planning.

Finally, patient preparation protocols varied considerably among the included studies, particularly regarding bowel cleansing prior to transvaginal ultrasound assessment of rectosigmoid endometriosis. While a few authors reported using dietary restrictions, laxatives, or enemas to improve visualization [18,32,33], most studies—including the series by Aas-Eng et al. [13,14,15]—performed TVUS without any preparation. This variability reflects the absence of an international consensus and the ongoing debate about the necessity and practicality of bowel cleansing in routine clinical settings.

### 3.3. Diagnostic Performance of TVUS for Uterosacral Ligament Involvement

**Figure 5:**
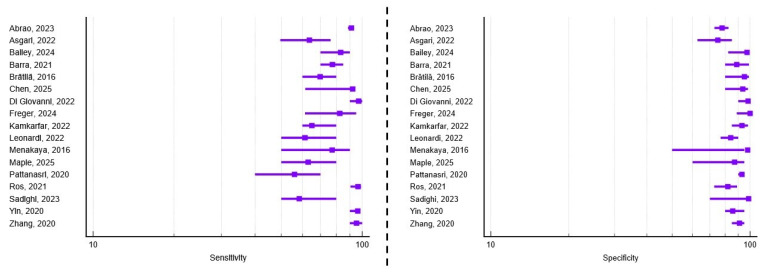
Forest plots of study-level diagnostic performance of transvaginal ultrasound for uterosacral ligament deep infiltrating endometriosis. Sensitivity (**left**) and specificity (**right**) are shown with squares representing point estimates and horizontal lines denoting 95% confidence intervals [11,16,18,19,20,21,22,24,26,28,30,31,33,36,37,40,41].

Out of the 30 studies included in this systematic review, 17 assessed the diagnostic accuracy of transvaginal ultrasound in detecting deep infiltrating endometriosis involving the uterosacral ligaments. Among these, 35.3% (n = 6) focused exclusively on this anatomical landmark. Regarding study design, eight were prospective, eight were retrospective, and one combined both methodologies. Most studies (76.47%) were conducted in single tertiary centers, ensuring consistent sonographic technique and standardized surgical correlation. The average number of patients per study was 387, ranging from 42 to 4983, with a mean age of 35.2 years across cohorts. Transvaginal probes between 5 and 12 MHz were predominantly used for high-resolution pelvic imaging. The average reported sensitivity and specificity were 78.07% (range 56–97%) and 90.49% (range 75.1–100%), respectively. A visual synthesis of these results is provided in the forest plots (Figure 5), which illustrate the variability in diagnostic performance across studies despite overall high values. Additional technical and methodological parameters related to ultrasound protocols, reporting systems, and diagnostic thresholds are detailed in Supplementary Table S3.

Among studies focusing on the uterosacral ligaments, the large prospective investigation by Di Giovanni et al. [24] included 4983 patients who underwent laparoscopic surgery for clinically suspected endometriosis. Each participant received a standardized preoperative ultrasound within one month of surgery, performed by an experienced sonographer using a combined transvaginal and transabdominal protocol with high-frequency 2D/3D probes. The uterosacral ligaments were systematically assessed along their cervical, intermediate, and sacral segments, with attention to adjacent parametrial regions and possible ureteral involvement. Deep endometriosis was diagnosed when irregular hypoechoic, poorly vascularized tissue disrupted the normal echogenic ligament pattern. Laparoscopy with histological confirmation served as the gold standard, yielding excellent diagnostic performance (sensitivity 97.3–98.3%, specificity 98.2–100%, overall accuracy 97.6%).

While most studies classified uterosacral ligament (USL) involvement according to anatomical location, Zhang et al. [42] proposed a distinct four-tiered morphological classification that integrates lesion shape and echogenic features. Type I lesions represented a thickened, hypoechoic root segment with disrupted continuity of the ligament, whereas type II lesions appeared as isolated, round or stellate hypoechoic nodules with variable margins. Type III lesions consisted of irregular, stripe-like hypoechoic extensions following the course of the ligament or spreading into adjacent parametrial regions, while type IV lesions combined features of the previous types and frequently involved both ligaments. This classification demonstrated high diagnostic performance, with a sensitivity of 95.3%, specificity of 90.9%, and overall accuracy of 94.1% for surgically confirmed USL involvement, suggesting its potential value for standardized reporting and routine clinical use.

To improve visualization of uterosacral ligament involvement, several studies explored adjunctive techniques designed to enhance image contrast during transvaginal ultrasound. In a prospective study, Chen et al. [22] demonstrated that introducing fluid into the pouch of Douglas significantly improved the delineation of USL margins and lesion morphology, thereby facilitating the detection of smaller nodules with limited infiltration. Compared with standard TVUS, the use of fluid instillation increased sensitivity from 61.5% to 92.3% and overall accuracy from 73.8% to 92.9%.

Reproducibility is a key prerequisite for the broader clinical implementation of ultrasound-based diagnostic techniques in deep infiltrating endometriosis, underscoring the importance of standardized and validated reporting systems. Despite this, relatively few studies have applied formalized protocols, reflecting ongoing heterogeneity in diagnostic approaches. Ros et al. [36] and Freger et al. [26] both employed protocols based on the International Deep Endometriosis Analysis (IDEA) consensus, reporting sensitivities of 96.9% and 82.6%, specificities of 87% and 100%, and overall accuracies of 89.5% and 92.6%, respectively, for detecting uterosacral ligament involvement. Conversely, Maple et al. [31] conducted their study in non-specialized imaging centers without a standardized reporting framework, achieving a moderate-to-good interobserver agreement (κ = 0.80) for USL lesions. However, such reproducibility remains largely operator-dependent in the absence of predefined sonographic criteria. The need for harmonization is further highlighted by the wide variation in ultrasound thresholds for defining USL involvement, with reported cut-off values ranging from 2 mm [24] to 7 mm [26], clustering around 5 mm in most studies [28,31,36].

### 3.4. Diagnostic Performance of TVUS for Pouch of Douglas Obliteration

**Figure 6:**
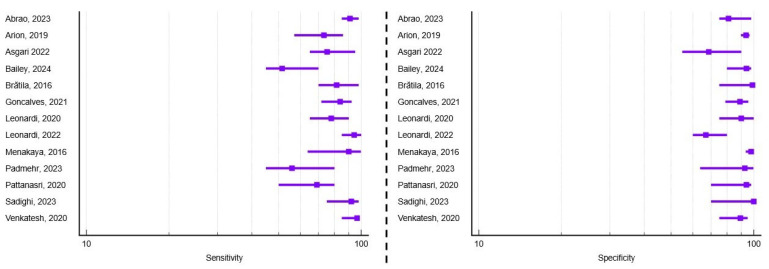
Forest plots of study-level diagnostic performance of transvaginal ultrasound for Pouch of Douglas obliteration. Sensitivity (**left**) and specificity (**right**) are shown with squares representing point estimates and horizontal lines denoting 95% confidence intervals [11,16,17,18,19,21,27,29,30,32,33,37,39].

Based on the inclusion criteria, 13 studies investigated the diagnostic accuracy of transvaginal ultrasound for identifying obliteration of the pouch of Douglas prior to surgery. Among these, 23.07% (n = 3) focused exclusively on this anatomical site. In terms of study design, six studies adopted a retrospective approach, six were prospective, while the study by Menakaya et al. [30] combined both approaches. Additionally, 69.23% (n = 9) of the studies included patients from a single tertiary diagnostic and treatment center. The average number of patients per study was 209, ranging from 42 to 878, and the mean age of the study population was 33.5 years. In terms of the type of surgical intervention, 23.07% (n = 3) of the papers reported performing laparoscopy with diagnostic biopsy, 7.69% (n = 1) employed electroablation of endometriotic nodules, while the remaining 69.23% (n = 9) planned complete excision of the identified lesions. The average reported sensitivity and specificity were 79.58% (range 51.5–96.6%) and 89.75% (range 66.9–100%), respectively. A visual synthesis of these findings is presented in Figure 6. Further methodological details, including reporting frameworks, operator experience, and diagnostic cut-off values used across studies, are provided in Supplementary Table S3.

Across the included studies, the sliding sign was the most widely used sonographic marker for detecting obliteration of the pouch of Douglas. This dynamic assessment involves applying gentle probe pressure to evaluate whether the anterior rectum glides freely over the posterior cervix and vaginal wall. A negative sliding sign—defined by restricted or absent movement—was consistently associated with pouch of Douglas obliteration caused by deep endometriosis-related adhesions. Reported diagnostic performance varied, with sensitivities ranging from 51.5% [19] to 96.6% [39], specificities from 78.8% [11] to 99.1% [27], and overall accuracy between 71.8% [18] and 96% [30], reaching 100% in smaller cohorts [29].

Transvaginal ultrasound has been shown to outperform manual pelvic examination in detecting obliteration of the pouch of Douglas. In a comparative study, Arion et al. [17] evaluated point-of-care TVUS against digital pelvic examination for the preoperative prediction of POD obliteration. A negative sliding sign on ultrasound demonstrated markedly higher sensitivity (73.2%) than palpation of a posterior fornix nodule during examination (24.4%), while both techniques achieved similarly high specificity (93.9% and 93.4%, respectively). The superior sensitivity of TVUS was attributed to the limited accessibility of high or small nodules during manual examination, as well as to cases of diffuse adhesions or inflammatory fibrosis lacking a discrete palpable lesion.

While most studies relied on standard transvaginal ultrasound techniques, a few implemented adjunctive methods to enhance diagnostic accuracy for posterior compartment endometriosis. Brătilă et al. [21] introduced a gel-based sonovaginography protocol that created a consistent acoustic window between the vaginal probe and posterior pelvic structures, improving visualization of the posterior cervix, uterosacral ligaments, rectovaginal septum, and pouch of Douglas. This modification, which eliminated the need for a second examiner and caused no patient discomfort, achieved a sensitivity of 81% and specificity of 94% for detecting pouch of Douglas lesions, closely matching surgical and histologic findings.

Additionally, Leonardi et al. [29] evaluated a saline-based modification of transvaginal ultrasound, in which the instillation of fluid into the pouch of Douglas created an acoustic window that improved visualization of adhesions and anatomical boundaries. This approach, particularly when combined with assessment of the sliding sign, enhanced diagnostic confidence in cases where standard TVUS offered limited detail. Reported diagnostic performance was high, with a sensitivity of 86.4%, specificity of 100%, and overall accuracy of 83.3% for detecting pouch of Douglas obliteration, confirmed by laparoscopy and histopathology.

Lastly, part of the heterogeneity observed among the included studies stemmed from differing definitions and classification systems for pouch of Douglas obliteration. Most authors adopted a binary distinction—obliterated versus non-obliterated—while others proposed more detailed grading schemes to better reflect the extent of adhesions. Abrao et al. [16] differentiated between partial and complete obliteration at laparoscopy, reporting that 42.5% of patients had partial and 32.5% complete involvement. Similarly, Gonçalves et al. [27] defined complete obliteration as the absence of visible peritoneum beneath the uterosacral ligaments, identifying this in 31% of cases. Building on these approaches, Padmehr et al. [32] and Arion et al. [17] employed three-tiered ultrasound-based models (normal, partial, and complete obliteration), observing complete obliteration in 40% and 30.5% of patients, respectively. This lack of uniformity in defining pouch of Douglas status complicates comparison of diagnostic accuracy across studies and limits the translation of findings into standardized clinical protocols.

## 4. Discussion

The present systematic review consolidates current evidence regarding the diagnostic performance of transvaginal ultrasound in evaluating deep infiltrating endometriosis of the posterior compartment, specifically involving the rectosigmoid colon, the uterosacral ligaments, and obliteration of the pouch of Douglas. Mean sensitivities and specificities reported in the included studies reached 83.05% and 90.53% for rectosigmoid disease, 78.07% and 90.49% for uterosacral ligament involvement, and 79.58% and 89.75% for pouch of Douglas obliteration, respectively. These findings underscore the utility of TVUS as a reliable, non-invasive modality for the preoperative evaluation of patients with suspected posterior compartment endometriosis.

Our findings align with previously published systematic reviews assessing preoperative findings of deep infiltrating endometriosis. Deslandes et al. [43] reported a wide range of diagnostic performance across anatomical sites, with sensitivities ranging from 10% to 98.9% and specificities from 46.1% to 100%, depending on lesion location, and overall accuracies between 75.7% and 97%. Likewise, Zhang et al. [41] demonstrated superior diagnostic performance of TVUS compared to physical examination, with pooled sensitivity and specificity estimates of 76% and 94%, respectively, and an area under the curve (AUC) of 0.92. While these reviews offer a comprehensive overview of TVUS performance across multiple compartments, the present study was specifically designed to evaluate its diagnostic accuracy within the posterior compartment—the most frequently affected region in extraovarian DIE. By narrowing our analysis to rectosigmoid involvement, uterosacral ligament lesions, and obliteration of the pouch of Douglas, this review provides a focused and clinically pertinent assessment of TVUS in the anatomical regions of highest surgical relevance.

The methodological quality of the included studies also requires consideration. According to the QUADAS-2 assessment, risks of bias were most apparent in patient selection, where preferential recruitment from referral centers and exclusion of women with prior surgery may have contributed to inflated diagnostic accuracy. Concerns were also frequent in the index test domain, as TVUS was often performed by expert operators who were not blinded to clinical or imaging findings, potentially overestimating diagnostic performance. Although laparoscopy with histology was generally applied as a robust reference standard, reporting was insufficiently detailed in some cohorts. Flow and timing were another area of uncertainty, with incomplete documentation of the interval between TVUS and surgical confirmation. With respect to applicability, concerns were most pronounced for the index test, as sonographers in the included studies were often highly experienced specialists, which may not reflect general gynecological practice. This concentration of expertise could have contributed to the high reported diagnostic accuracy.

These methodological concerns align with broader contextual limitations. Most of the included studies were performed in tertiary referral centers with expert examiners and a high prevalence of endometriosis, conditions that differ from routine gynecological practice. Although TVUS demonstrates high accuracy in expert hands, more modest results have been reported in non-specialist settings, underscoring the operator dependence of the technique. This variability highlights the need for further validation in general gynecological practice.

A considerable degree of heterogeneity characterized the scanning protocols for TVUS in posterior compartment deep infiltrating endometriosis, limiting comparability across studies and hindering the establishment of standardized clinical practice. One major source of variability relates to patient bowel preparation, as some protocols recommend dietary restrictions, laxatives, or rectal enemas to improve visualization of rectosigmoid lesions, while many studies omit bowel preparation altogether. Additional inconsistency arises from the adjunctive use of techniques such as gel instillation for sonovaginography or saline infusion into the pouch of Douglas, which are applied selectively rather than systematically, thereby limiting reproducibility. Although efforts have been made to address these issues through the introduction of standardized approaches—most notably the IDEA consensus [11]—these frameworks have not yet achieved universal adoption. The absence of widespread consensus on preparation, reporting, and classification highlights the ongoing need for harmonization to enhance reproducibility and ensure the reliable integration of TVUS into routine preoperative assessment. While subgroup or meta-regression analyses could have further clarified the impact of these factors, the inconsistent reporting and limited number of comparable studies precluded such quantitative exploration in this review. This limitation reinforces the importance of standardized methodologies in future research to allow for meaningful pooled subgroup analyses. Moreover, definitions of uterosacral ligament involvement and pouch of Douglas obliteration varied substantially across studies, contributing additional heterogeneity and limiting the generalizability of pooled interpretations.

Despite these inconsistencies, transvaginal ultrasound is reported as one of the first-line non-invasive diagnostic modalities for posterior compartment deep infiltrating endometriosis. Multiple systematic reviews and meta-analyses have consistently shown that both TVUS and MRI achieve high diagnostic accuracy, with the latter demonstrating sensitivities of 83% to 90% and specificities of 95% to 96% [44].

Current international guidelines recommend transvaginal ultrasound as the initial imaging modality, reserving MRI for cases in which ultrasound is inconclusive, clinical suspicion remains high, or comprehensive preoperative mapping of complex or extrapelvic disease is required. This hierarchy is supported not only by comparable diagnostic performance but also by practical considerations of cost-effectiveness and accessibility. Dedicated MRI protocols for endometriosis require 30–45 min of acquisition time and may cost up to USD 700, which limits their availability in many healthcare settings [45]. Moreover, accessibility is often constrained by long waiting times for non-urgent outpatient MRI, which may extend from 8 to 12 weeks in Norway and approximately 3 months in Canada, with significant regional variation [46]. By contrast, TVUS is inexpensive, widely accessible, routinely performed by gynecologists during standard consultations, and can be seamlessly integrated into the initial diagnostic work-up. Although MRI offers standardized imaging and is less dependent on operator expertise—an advantage in non-specialist environments—its higher cost, longer acquisition, and reduced accessibility restrict its role to selected scenarios. These findings support current guideline recommendations of TVUS as the first-line imaging modality for posterior compartment endometriosis in routine clinical practice, while also underscoring the need for greater standardization of scanning protocols and reporting frameworks. To enhance clinical applicability, Table 2 summarizes the scenarios in which TVUS is generally sufficient as a stand-alone modality and those where MRI may provide added diagnostic value, in accordance with the American College of Radiology guidelines [47].

A key step toward reducing this variability has been the introduction of standardized reporting frameworks, most notably the International Deep Endometriosis Analysis (IDEA) consensus [11]. The IDEA group proposed a systematic approach to transvaginal ultrasound evaluation with standardized terminology and examination steps aimed at minimizing heterogeneity and improving reproducibility. Subsequent prospective validation, including a multicenter pilot study of 273 women across eight centers, confirmed that adherence to the IDEA protocol yields high diagnostic performance for deep endometriosis, with a sensitivity of 88–90% and a specificity of 76–79%, alongside positive predictive values exceeding 90%. These results compare favorably with pooled sensitivities of around 79% reported in earlier reviews conducted before IDEA implementation, underscoring the added value of structured protocols in enhancing diagnostic confidence while maintaining generalizability. Beyond improving reproducibility, systematic mapping according to IDEA facilitates surgical planning by accurately localizing rectosigmoid nodules and anticipating the need for advanced procedures such as bowel resection. Importantly, proficiency in applying the IDEA methodology improves with experience; studies suggest that diagnostic accuracy in detecting rectal deep endometriosis increases after approximately 40 supervised examinations, highlighting the relevance of structured training and operator learning curves for broader implementation.

Beyond its diagnostic performance, TVUS also carries significant clinical value in preoperative planning. Ultrasound-based staging systems, particularly the Ultrasound-Based Endometriosis Staging System (UBESS), have demonstrated remarkable accuracy in anticipating the complexity of laparoscopic surgery. In a large prospective cohort of 157 women, the UBESS achieved an overall accuracy of 96.7%, with a Cohen’s kappa of 0.94, indicating near-perfect agreement with the RANZCOG/AGES surgical skill classification system [48]. Stage-specific performance was particularly strong: UBESS II predicted moderate-complexity (Levels 3–4) laparoscopies with 96.7% accuracy, while UBESS III anticipated high-complexity (Level 6) procedures with 97.3% accuracy. These findings extend earlier reports by Menakaya et al. [30], confirming that structured TVUS staging reliably informs surgical triage and supports multidisciplinary planning in advanced disease.

Emerging imaging modalities may further refine the diagnostic pathway for posterior compartment endometriosis. Three-dimensional transvaginal ultrasound (3D US) has demonstrated superior diagnostic accuracy compared with conventional two-dimensional US, with higher AUC values (0.891 vs. 0.789, *p* = 0.0193) and improved sensitivity and specificity for non-intestinal posterior lesions, while maintaining comparable performance for intestinal involvement. Its reproducibility is substantial even among operators with varying expertise, and it can provide valuable details for surgical planning and multidisciplinary management. However, its broader use is constrained by equipment availability and operator training, and it does not reliably detect superficial peritoneal endometriosis [49]. Diffusion-weighted imaging (DWI) within MRI protocols offers complementary information by highlighting the low apparent diffusion coefficient (ADC) values characteristic of the dense fibrotic tissue typical of deep infiltrating endometriosis. This makes DWI particularly useful for lesion identification and confirmation across pelvic sites, including the posterior compartment. Nonetheless, DWI is not a stand-alone diagnostic tool; its role lies in enhancing lesion conspicuity and diagnostic confidence when interpreted alongside standard T1- and T2-weighted sequences, rather than replacing them [50].

In summary, transvaginal ultrasound emerges from the current evidence as a reliable, accessible, and cost-effective tool for diagnosing posterior compartment deep infiltrating endometriosis, particularly when performed by experienced operators. Its diagnostic performance has been reported within a similar range to that of MRI in previous systematic reviews, supporting its use as a valuable first-line modality. Nevertheless, heterogeneity in methodology, operator expertise, and reporting standards remains considerable, and further validation in general gynecological practice is warranted. In line with international guidelines, TVUS can be considered the preferred initial imaging approach, with MRI reserved for inconclusive cases or for comprehensive mapping of complex or extrapelvic disease. Ongoing efforts to refine methodology, standardize protocols, and improve reproducibility are essential to optimize its broader clinical implementation.

## 5. Limitations

While this review provides a focused evaluation of posterior compartment disease and integrates the available evidence in a structured manner, certain limitations should be acknowledged. Firstly, a formal meta-analysis could not be performed due to the substantial heterogeneity in study methodologies and outcome definitions. At the review level, additional limitations include the absence of protocol registration and the exclusion of non-English studies, which may have introduced bias. Although the review adhered to PRISMA 2020 guidelines, the more specialized PRISMA-DTA framework might have provided further detail specific to diagnostic accuracy studies, and its absence should be considered a limitation. Secondly, several studies were limited by small sample sizes, retrospective designs, and single-center settings, which may introduce bias and restrict the strength of the conclusions. Thirdly, as most investigations originated from tertiary referral centers and were conducted by expert sonographers, the findings may not be fully generalizable to routine gynecological practice. Finally, data on interobserver variability and operator learning curves were scarce, despite these factors being particularly important for supporting the wider clinical implementation of TVUS. Moreover, definitional heterogeneity—particularly in the classification of uterosacral ligament involvement and pouch of Douglas obliteration—further constrained comparability across studies and may have influenced reported diagnostic performance. Additionally, as most included studies were conducted in tertiary referral centers with a high disease prevalence and expert operators, the findings may not be fully generalizable to routine gynecological practice.

## 6. Conclusions

Transvaginal ultrasound demonstrates high diagnostic accuracy for posterior compartment deep infiltrating endometriosis and, in accordance with international guidelines, should be regarded as a reliable first-line imaging modality in expert hands. However, its performance is operator-dependent, and the generalizability of results from tertiary referral centers to routine gynecological settings remains limited. Standardization of scanning protocols and reporting systems is therefore a priority for enhancing reproducibility and facilitating broader implementation. MRI retains an important complementary role in cases with inconclusive ultrasound findings or when comprehensive mapping of complex or extrapelvic disease is required.

## Figures and Tables

**Table 1:** Characteristics and main findings of studies on transvaginal ultrasound for posterior compartment endometriosis.

No.	First Author and Year	Country	Study Design	Sample Size	Mean Age (Years)	Transducer Frequency (MHz)	Type of Surgical intervention	RS Performance (%)	USL Performance (%)	PDO Performance (%)
1.	Aas-Eng, 2020 [13]	Austria Australia Norway	Prospective Multicenter	147	35.3	5–9	Bowel resection	Se = 74 Sp = 86	N/A	N/A
2.	Aas-Eng, 2021 [14]	Austria Australia Norway	Prospective Multicenter	207	35.7	5–9	Bowel resection	Se = 75 Sp = 87	N/A	N/A
3.	Aas-Eng, 2023 [15]	Norway	Prospective Single-center	75	38.3	5–9	Bowel resection	Se = 85 Sp = 89	N/A	N/A
4.	Abrao, 2023 [16]	Brazil Italy Spain	Retrospective Multicenter	878	37.3	4–9	Bowel resection Electroablation	Se = 96.1 Sp = 97 Acc = 96.7	Se = 91.1 Sp = 77.9 Acc = 86.5	Se = 93 Sp = 81 Acc = 85.9
5.	Arion, 2019 [17]	Canada	Prospective Single-center	269	34.4	5–9	Lesion excision	N/A	N/A	Se = 73.2 Sp = 93.9
6.	Asgari, 2022 [18]	Iran	Retrospective Single-center	119	35.4	5–9	Bowel resection/shaving Lesion excision	Se = 52.9 Sp = 94.1 Acc = 73.5	Se = 63.6 Sp = 75.1 Acc = 69.3	Se = 75.1 Sp = 68.6 Acc = 71.8
7.	Bailey, 2024 [19]	UK	Retrospective Single-center	100	35.2	5–9	Lesion excision	N/A	Se = 83.3 Sp = 97.4 Acc = 81.6	Se = 51.5 Sp = 94 Acc = 87.4
8.	Barra, 2021 [20]	Brazil	Prospective Single-center	281	36.8	6–9	Bowel resection Lesion excision	Se = 82 Sp = 98.5 Acc = 93.6	Se = 77.6 Sp = 88.8 Acc = 82.6	N/A
9.	Brătilă, 2016 [21]	Romania	Prospective Multicenter	193	32	7.5	Bowel shaving Lesion excision	Se = 91 Sp = 95.5	Se = 69.7 Sp = 95	Se = 81.5 Sp = 99
10.	Chen, 2025 [22]	China	Prospective Single-center	42	36.4	6–12	Laparoscopy Biopsy	N/A	Se = 92.3 Sp = 93.8 Acc = 92.9	N/A
11.	Di Giovanni, 2018 [23]	Italy	Prospective Single-center	328	34.9	5–9	Bowel resection/shaving	Se = 91.4 Sp = 100 Acc = 93	N/A	N/A
12.	Di Giovanni, 2022 [24]	Italy	Prospective Single-center	4983	Not reported *	5–9	Lesion excision	N/A	Se = 97 Sp = 98	N/A
13.	Ferrero, 2019 [25]	Italy	Prospective Single-center	262	34.1	6–12	Bowel resection	Se = 88.1 Sp = 95.8 Acc = 92.3	N/A	N/A
14.	Freger, 2024 [26]	Canada	Prospective Single-center	54	35.2	4–9	Laparoscopy Biopsy	N/A	Se = 82.6 Sp = 100 Acc = 92.6	N/A
15.	Goncalves, 2021 [27]	Brazil	Prospective Single-center	120	33.6	6–12	Bowel resection/shaving Lesion excision	Se = 96.2 Sp = 98.5 Acc = 97.1	N/A	Se = 83.9 Sp = 89.1 Acc = 86.7
16.	Kamkarfar, 2022 [28]	Iran	Prospective Single-center	80	34.4	5–9	Lesion excision Biopsy	N/A	Se = 65 Sp = 93	N/A
17.	Leonardi, 2020 [29]	Australia	Prospective Single-center	42	Not reported *	5–9	Laparoscopy Biopsy	N/A	N/A	Se = 77.7 Sp = 100 Acc = 83.3
18.	Leonardi, 2022 [11]	Australia Austria Germany Israel Italy Spain	Prospective Multicenter	273	34	Not reported individually for each center *	Bowel resection/shaving Lesion excision Biopsy only	Se = 96 Sp = 86.2 Acc = 89.8	Se = 61.2 Sp = 84.2 Acc = 74.6	Se = 94.2 Sp = 66.9 Acc = 77.3
19.	Menakaya, 2016 [30]	Australia	Combined Multicenter	192	23.7	7.5	Laparoscopy Biopsy	Se = 83.3 Sp = 90.8 Acc = 89.4	Se = 77.3 Sp = 97.8 Acc = 92	Se = 90.2 Sp = 98 Acc = 96
20.	Maple, 2025 [31]	Australia	Retrospective Single-center	42	33	3–11	Laparoscopy Biopsy	N/A	Se = 63 Sp = 87	N/A
21.	Padmehr, 2023 [32]	Iran	Retrospective Single-center	170	34.4	Not reported *	Bowel resection Lesion excision	Se = 69 Sp = 88.3 Acc = 84.8	N/A	Se = 56 Sp = 92.8 Acc = 88.9
22.	Pattanasri, 2020 [33]	Australia	Retrospective Single-center	119	36	Not reported *	Bowel resection Lesion excision	Se = 71 Sp = 98 Acc = 85	Se = 56 Sp = 93 Acc = 75	Se = 69 Sp = 94 Acc = 82
23.	Reid, 2018 [34]	Australia	Prospective Multicenter	376	Not reported *	7.5	Bowel resection	Se = 86.8 Sp = 92.3 Acc = 87	N/A	N/A
24.	Ros, 2017 [35]	Spain	Retrospective Single-center	40	36.8	5–9	Bowel resection	Se = 73 Sp = 88 Acc = 82.5	N/A	N/A
25.	Ros, 2021 [36]	Spain	Prospective Single-center	172	38.3	5–9	Laparoscopy Biopsy	N/A	Se = 96.6 Sp = 82.1 Acc = 89.5	N/A
26.	Sadighi, 2023 [37]	Iran	Retrospective Single-center	110	37.2	Not reported *	Lesion excision Biopsy	N/A	Se = 58.3 Sp = 98.7 Acc = 89.5	Se = 92 Sp = 100 Acc = 93.3
27.	Sloss, 2022 [38]	Australia	Retrospective Single-center	135	36.7	5–9	Bowel resection/shaving Biopsy only	Se = 93.6 Sp = 50	N/A	N/A
28.	Venkatesh, 2020 [39]	India	Prospective Single-center	136	29.3	4–8	Laparoscopy Biopsy	N/A	N/A	Se = 96.6 Sp = 89.5 Acc = 94.1
29.	Yin, 2020 [40]	China	Retrospective Single center	198	35.3	5–9	Lesion resection	Se = 94.4 Sp = 94.6 Acc = 94.9	Se = 96.4 Sp = 85.7 Acc = 94.9	N/A
30.	Zhang, 2020 [41]	China	Retrospective Single-center	118	35.2	5–9	Laparoscopy Biopsy	N/A	Se = 95.3 Sp = 90.9 Acc = 94.1	N/A

MHz = megahertz; RS = rectosigmoid endometriosis; USL = uterosacral ligament involvement; PDO = pouch of Douglas obliteration; Se = sensitivity; Sp = specificity; Acc = accuracy; N/A = not applicable. * Missing values reflect incomplete reporting in the source studies rather than omission during data extraction.

**Table 2:** Recommended imaging modality by clinical scenario, based on the American College of Radiology guidelines.

Clinical Scenario	Preferred Modality	Notes
Initial evaluation of suspected endometriosis	TVUS	Widely available, cost-effective, performed in routine gynecology visits
Assessment of rectosigmoid, uterosacral ligament, or pouch of Douglas involvement	TVUS	High diagnostic accuracy when performed by trained operators
Inconclusive or negative TVUS but high clinical suspicion	MRI	Provides complementary mapping
Preoperative assessment of complex/multifocal disease	MRI	Helpful for surgical planning, particularly with extrapelvic extension
Centers without specialized ultrasound expertise	MRI	Ensures comprehensive mapping

TVUS = transvaginal ultrasound; MRI = magnetic resonance imaging.

## Data Availability

No new data were created or analyzed in this study. Data sharing is not applicable to this article.

## Supplementary Materials

The following supporting information can be downloaded at: https://www.mdpi.com/article/10.3390/healthcare13202548/s1. Table S1: PRISMA 2020 Checklist; Table S2: QUADAS-2 risk of bias and applicability assessments with per-study justifications.; Table S3: Summary of transvaginal ultrasound protocols and diagnostic frameworks used across the studies included in this systematic review. Reference [51] is cited in the Supplementary Materials.

## Abbreviations

The following abbreviations are used in this manuscript:

Acc	Accuracy
ADC	Apparent Diffusion Coefficient
AUC	Area Under the Curve
DIE	Deep Infiltrating Endometriosis
DWI	Diffusion-Weighted Imaging
ESHRE	European Society of Human Reproduction and Embryology
IDEA	International Deep Endometriosis Analysis
MRI	Magnetic Resonance Imaging
N/A	Not Applicable
NICE	National Institute for Health and Care Excellence
PIRT	Participants, Index test, Reference standard, Target condition
PDO	Pouch of Douglas Obliteration
PRISMA	Preferred Reporting Items for Systematic Reviews and Meta-Analyses
QUADAS-2	Quality Assessment of Diagnostic Accuracy Studies, Version 2
RS	Rectosigmoid Endometriosis
Se	Sensitivity
SOGC	Society of Obstetricians and Gynecologists of Canada
Sp	Specificity
TVUS	Transvaginal Ultrasound
UBESS	Ultrasound-Based Endometriosis Staging System
USL	Uterosacral Ligament
