# Supplementary material for: The Diagnostic Performance of Transvaginal Ultrasound for Posterior Compartment Endometriosis Compared to Laparoscopic and Histopathological Findings: A Systematic Review
Source: Healthcare (Basel). 2025 Oct 10;13(20):2548. doi: 10.3390/healthcare13202548 (PMC12563166; doi:10.3390/healthcare13202548)
Supplement: Supplementary file 1 [file healthcare-13-02548-s001.zip › healthcare-3871171-supplementary.pdf]

PRISMA 2020 Checklist

Section and Topic	Item #	Checklist item	Location where item is reported
**TITLE**			
Title	1	Identify the report as a systematic review.	Page 1: Title and paper type
**ABSTRACT**			
Abstract	2	See the PRISMA 2020 for Abstracts checklist.	Page 1: Abstract
**INTRODUCTION**			
Rationale	3	Describe the rationale for the review in the context of existing knowledge.	Page 2: Introduction
Objectives	4	Provide an explicit statement of the objective(s) or question(s) the review addresses.	Page 2: Introduction, last paragraph
**METHODS**			
Eligibility criteria	5	Specify the inclusion and exclusion criteria for the review and how studies were grouped for the syntheses.	Page 3: Materials and Methods, 2nd and 3rd paragraph, plus bullet list
Information sources	6	Specify all databases, registers, websites, organisations, reference lists and other sources searched or consulted to identify studies. Specify the date when each source was last searched or consulted.	Page 3: Materials and Methods, 1st paragraph
Search strategy	7	Present the full search strategies for all databases, registers and websites, including any filters and limits used.	Page 3: Materials and Methods, 1st paragraph
Selection process	8	Specify the methods used to decide whether a study met the inclusion criteria of the review, including how many reviewers screened each record and each report retrieved, whether they worked independently, and if applicable, details of automation tools used in the process.	Page 3: Materials and Methods, 1st paragraph
Data collection process	9	Specify the methods used to collect data from reports, including how many reviewers collected data from each report, whether they worked independently, any processes for obtaining or confirming data from study investigators, and if applicable, details of automation tools used in the process.	Page 3: Materials and Methods, 1st paragraph
Data items	10a	List and define all outcomes for which data were sought. Specify whether all results that were compatible with each outcome domain in each study were sought (e.g. for all measures, time points, analyses), and if not, the methods used to decide which results to collect.	Page 4: Materials and Methods, 5th paragraph
	10b	List and define all other variables for which data were sought (e.g. participant and intervention characteristics, funding sources). Describe any assumptions made about any missing or unclear information.	Page 4: Materials and Methods, 5th paragraph
Study risk of bias assessment	11	Specify the methods used to assess risk of bias in the included studies, including details of the tool(s) used, how many reviewers assessed each study and whether they worked independently, and if applicable, details of automation tools used in the process.	Pages 4 : Materials and Methods, 6th paragraph
Effect measures	12	Specify for each outcome the effect measure(s) (e.g. risk ratio, mean difference) used in the synthesis or presentation of results.	Page 4: Materials and Methods, 5th paragraph

Section and Topic	Item #	Checklist item	Location where item is reported
Synthesis methods	13a	Describe the processes used to decide which studies were eligible for each synthesis (e.g. tabulating the study intervention characteristics and comparing against the planned groups for each synthesis (item #5)).	Pages 4 – 5: Materials and Methods, 6th paragraph
	13b	Describe any methods required to prepare the data for presentation or synthesis, such as handling of missing summary statistics, or data conversions.	Pages 4 – 5: Materials and Methods, 7th paragraph
	13c	Describe any methods used to tabulate or visually display results of individual studies and syntheses.	Pages 4 – 5: Materials and Methods, 6th paragraph
	13d	Describe any methods used to synthesize results and provide a rationale for the choice(s). If meta-analysis was performed, describe the model(s), method(s) to identify the presence and extent of statistical heterogeneity, and software package(s) used.	Pages 4 – 5: Materials and Methods, 6th paragraph
	13e	Describe any methods used to explore possible causes of heterogeneity among study results (e.g. subgroup analysis, meta-regression).	Pages 4 – 5: Materials and Methods, 6th paragraph
	13f	Describe any sensitivity analyses conducted to assess robustness of the synthesized results.	Pages 4 – 5: Materials and Methods, 6th paragraph
Reporting bias assessment	14	Describe any methods used to assess risk of bias due to missing results in a synthesis (arising from reporting biases).	Pages 4 – 5: Materials and Methods, 6th paragraph
Certainty assessment	15	Describe any methods used to assess certainty (or confidence) in the body of evidence for an outcome.	Not applicable
**RESULTS**			
Study selection	16a	Describe the results of the search and selection process, from the number of records identified in the search to the number of studies included in the review, ideally using a flow diagram.	Page 5: Results, 1st and 2nd paragraph, Figure 1
	16b	Cite studies that might appear to meet the inclusion criteria, but which were excluded, and explain why they were excluded.	Page 5: Results, 1st and 2nd paragraph
Study characteristics	17	Cite each included study and present its characteristics.	Pages 5 – 8: Table 1
Risk of bias in studies	18	Present assessments of risk of bias for each included study.	Page 5: Results, 3rd paragraph and Figures 2 and 3

Section and Topic	Item #	Checklist item	Location where item is reported
Results of individual studies	19	For all outcomes, present, for each study: (a) summary statistics for each group (where appropriate) and (b) an effect estimate and its precision (e.g. confidence/credible interval), ideally using structured tables or plots.	Pages 5 – 8: Table 1
Results of syntheses	20a	For each synthesis, briefly summarise the characteristics and risk of bias among contributing studies.	Page 8 – 9: Figures 2 and 3
	20b	Present results of all statistical syntheses conducted. If meta-analysis was done, present for each the summary estimate and its precision (e.g. confidence/credible interval) and measures of statistical heterogeneity. If comparing groups, describe the direction of the effect.	Not applicable
	20c	Present results of all investigations of possible causes of heterogeneity among study results.	Results: Last paragraphs from sections 3.2, 3.3 and 3.4
	20d	Present results of all sensitivity analyses conducted to assess the robustness of the synthesized results.	Not applicable
Reporting biases	21	Present assessments of risk of bias due to missing results (arising from reporting biases) for each synthesis assessed.	Not applicable
Certainty of evidence	22	Present assessments of certainty (or confidence) in the body of evidence for each outcome assessed.	Not applicable
**DISCUSSION**			
Discussion	23a	Provide a general interpretation of the results in the context of other evidence.	Page 13: Discussion, 1st and 2nd paragraphs
	23b	Discuss any limitations of the evidence included in the review.	Page 14: Limitations
	23c	Discuss any limitations of the review processes used.	Page 14: Limitations
	23d	Discuss implications of the results for practice, policy, and future research.	Pages 13 – 14: Discussion, 3rd and 4th paragraph
**OTHER INFORMATION**			
Registration and protocol	24a	Provide registration information for the review, including register name and registration number, or state that the review was not registered.	Not applicable
	24b	Indicate where the review protocol can be accessed, or state that a protocol was not prepared.	Not applicable
	24c	Describe and explain any amendments to information provided at registration or in the protocol.	Not applicable
Support	25	Describe sources of financial or non-financial support for the review, and the role of the funders or sponsors in the review.	Not applicable
Competing interests	26	Declare any competing interests of review authors.	None
Availability of data, code and other materials	27	Report which of the following are publicly available and where they can be found: template data collection forms; data extracted from included studies; data used for all analyses; analytic code; any other materials used in the review.	All data extracted and analyzed in this review are contained within the article

From: Page MJ, McKenzie JE, Bossuyt PM, Boutron I, Hoffmann TC, Mulrow CD, et al. The PRISMA 2020 statement: an updated guideline for reporting systematic reviews. BMJ 2021;372:n71. doi: 10.1136/bmj.n71.
This work is licensed under CC BY 4.0. To view a copy of this license, visit https://creativecommons.org/licenses/by/4.0/

Supplementary Table S1. PRISMA 2020 Checklist

	Risk of Bias					Applicability Concerns			
	Patient Selection	Index Test	Reference Standard	Flow and Timing	Key Notes	Patient Selection	Index Test	Reference Standard	Key Notes
Aas-Eng, 2020 [13]	Low	Unclear	Low	Unclear	Consecutive patients; blinding unclear; laparoscopy reference; timing not reported	Low	Unclear	Low	Representative population; TVS protocol unclear details; reference appropriate
Aas-Eng, 2021 [14]	Low	Unclear	Low	Unclear	Consecutive recruitment ; blinding of index test not fully reported; surgical specimen measurement as reference standard; timing between TVS and surgery not specified	Low	Unclear	Low	Population representative of rectosigmoid DE surgery; TVS protocol based on IDEA consensus; reference standard appropriate; no major concerns.
Aas-Eng, 2023 [15]	Low	Unclear	Low	Low	Consecutive recruitment; single-center (unclear generalizability); blinding of imaging examiners reported; IOM with rectal probe as reference; no inter/intraobserver variability	Low	Unclear	Low	Population: symptomatic rectosigmoid DE surgery (representative). TVS performed by experienced examiner; MRI protocols partly heterogeneous (minor concern). Reference standard appropriate. No major applicability issues
Abrao, 2023 [16]	Low	Unclear	Low	High	Retrospective design; consecutive surgical cases from 3 centers. Index test by expert sonographers, blinded to surgery but aware of	Low	Unclear	Low	Large multicenter surgical population, mostly advanced-stage disease (potential spectrum bias). TVS protocols

					symptoms. Laparoscopy with histology as reference. Exclusion of 21% with incomplete data may bias results				standardized and clinically relevant. Reference standard appropriate. Minor applicability concerns
Arion, 2019 [17]	Low	High	Low	Low	Prospective; blinding absent; laparoscopy ref.; single-center, limited generalizability	Low	Unclear	Low	Tertiary referral population; TVUS sliding sign relevant; reference appropriate
Asgari, 2022 [18]	High	High	Low	Low	Retrospective; referral center, high prevalence; sonographer aware of clinical exam; laparoscopy + histology ref.; limited rare sites	Unclear	Low	Low	Tertiary referral cohort, advanced cases; IDEA protocol used; expert examiner; limited generalizability to general population
Bailey, 2024 [19]	High	Unclear	Low	Unclear	Retrospective; single expert sonographer; surgeons not blinded; histology/laparoscopy ref.; high false-negatives for SE	Low	Unclear	Low	Tertiary referral cohort; focus on POD-SE; highly trained examiner; limited generalizability to routine practice
Barra, 2021 [20]	Low	Low	Low	Low	Prospective multicenter; consecutive patients; blinding of US vs. surgery unclear; laparoscopy ref.; variable examiner expertise	Low	Low	Low	Mixed population with suspected DE; TVUS and MRI protocols varied; reference standard appropriate; minor applicability concerns.
Brătilă, 2016 [21]	Low	Unclear	Low	Low	Prospective multicenter; highly symptomatic patients (spectrum bias); same operators for TVS/SVG and aware before surgery;	Unclear	Low	Low	Tertiary referral population; SVG with gel less common in routine; reference standard appropriate;

					laparoscopy + histology ref.				limited generalizability
Chen, 2025 [22]	Unclear	Low	Low	Low	Single-center; patient selection not clearly described; standardized index test; laparoscopy + histology reference; timing adequate	Unclear	Low	Low	Referral population, unclear representativeness; index test protocol adequate; reference standard appropriate.
Di Giovanni, 2018 [23]	Unclear	High	Low	Low	Prospective; single expert sonographer; no blinding; inclusion only of surgical bowel resections; reference = laparoscopy + histology.	High	Unclear	Low	Specialized center; highly experienced operator; limited generalizability
Di Giovanni, 2022 [24]	Unclear	High	Low	Low	Retrospective; referral population, very high prevalence; single expert examiner (>10,000 scans); surgeons aware of US findings (no blinding, high); laparoscopy + histology ref	High	Unclear	Low	Specialized referral center; nearly 5000 surgical cases, severe DIE; expert-only setting; not generalizable to routine practice.
Ferrero, 2019 [25]	Low	Low	Unclear	Low	Prospective consecutive cohort; patient selection low risk; blinding of US vs. surgery not clearly reported; laparoscopy + histology ref.; timing acceptable.	Unclear	Unclear	Low	Referral surgical population; representativeness unclear; index test protocol not fully detailed; reference appropriate.
Freger, 2024 [26]	Unclear	Unclear	Unclear	Unclear	Consecutive patients; single expert sonologist & surgeon; blinding absent; laparoscopy ± histology ref.; small sample (54)	Unclear	Unclear	Unclear	Specialized tertiary center; expert-only; technique not yet validated in wider settings

Goncalves, 2021 [27]	Unclear	Low	Unclear	Low	Patient selection unclear; index test standardized; reference standard not fully detailed; timing acceptable	Unclear	Unclear	Unclear	Referral population; index protocol not fully described; applicability uncertain
Kamkarfar, 2022 [28]	Unclear	Low	Unclear	Low	Cross-sectional; consecutive symptomatic patients; single expert examiner; blinding unclear; laparoscopy + histology as reference.	Unclear	Low	Unclear	Referral hospital population; TVS performed by highly skilled operator; good reference standard; generalizability limited
Leonardi, 2020 [29]	Unclear	Unclear	Unclear	Low	Multicenter registry; patient selection unclear; imaging protocols not uniform; blinding not reported; laparoscopy + histology ref	Unclear	Unclear	Low	International cohort; heterogeneous examiners; index test protocols variable; reference standard adequate.
Leonardi, 2022 [11]	Low	Unclear	Unclear	Low	Multicenter prospective; broad inclusion; imaging methods not standardized; reference = laparoscopy ± histology.	Low	Unclear	Low	International surgical cohort; variable examiner expertise (unclear); reference standard consistent and appropriate (low)
Menakaya, 2016 [30]	Unclear	Unclear	Unclear	Unclear	Patient selection unclear; index test blinding not reported; reference standard unclear; timing not specified	Unclear	Unclear	Low	Referral population; index protocol not well described; reference applicability uncertain
Maple, 2025 [31]	Unclear	Unclear	High	Unclear	Consecutive patients; blinding unclear; laparoscopy ref.; timing not reported	Unclear	Unclear	High	Representative population; TVS protocol adequate; reference

									appropriate.
Padmehr, 2023 [32]	Unclear	High	High	Low	Retrospective cross-sectional; referral population; surgeons aware of TVS findings (no blinding, high risk); three expert radiologists with access to clinical data (operator-dependent, high risk); laparoscopic + histology reference standard (low risk).	High	High	Low	Referral center, high disease prevalence, expert-only setting; limited generalizability to routine practice.
Pattanasri, 2020 [33]	Unclear	High	Low	Unclear	Retrospective cohort with possible selection bias; specialist vs community TVUS without clear blinding; laparoscopy with histology used as gold standard; timing interval not always specified.	Unclear	Unclear	Low	Referral private setting with high prevalence; operator expertise (specialist vs community) limits generalizability, though reference standard appropriate.
Reid, 2018 [34]	Unclear	Low	Unclear	Low	Prospective design; unclear patient selection and blinding of index test; laparoscopy with histology reference standard; sample size moderate	Unclear	Low	Low	Population representative; TVS protocol not fully described; reference standard appropriate.
Ros, 2017 [35]	Unclear	Low	Low	Unclear	Prospective design; consecutive recruitment; blinding of index test not clearly reported; laparoscopy ± histology as reference standard; flow and timing partly	Unclear	Low	Low	Study population representative; TVS protocol insufficiently detailed; reference standard appropriate.

					unclear				
Ros, 2021 [36]	Unclear	Low	Low	Low	Prospective single-center design; consecutive patients; expert sonographers blinded to clinical history; laparoscopic + histological reference standard.	Low	Low	Low	Referral tertiary center with high disease prevalence; specialized setting may limit generalizability to routine practice.
Sadighi, 2023 [37]	Unclear	Low	Low	Low	Prospective design; consecutive recruitment; blinding of index test not clearly reported; laparoscopy with histology reference standard; low risk across domains.	Low	Low	Low	Representative surgical population; clear TVS protocol; appropriate reference standard; findings generalizable
Sloss, 2022 [38]	High	Unclear	Low	High	Retrospective single-center analysis; unclear patient selection; index tests performed by expert sonologist/radiologist with access to clinical data (operator-dependent); histology reference standard; long imaging-to-surgery interval.	Unclear	Low	Low	Tertiary referral population with high prevalence of rectosigmoid DIE; specialist-performed imaging limits generalizability; reference standard appropriate
Venkatesh, 2020 [39]	Unclear	Unclear	Unclear	Low	Prospective observational; consecutive recruitment; single experienced observer (operator-dependent); blinding of index test unclear; laparoscopy reference standard	Low	Low	Unclear	Tertiary referral center; population mostly young and nulliparous with infertility; TVS protocol described but limited generalizability outside expert setting
Yin, 2020 [40]	Unclear	Unclear	Low	Low	Retrospective, single-center design;	Low	Low	Low	Study population representative

					consecutive patients with suspected DIE; blinding of examiner reported; laparoscopic–histology reference standard; limited cases for bladder, ureter, broad ligament.				(symptomatic women undergoing surgery); experienced operator; TVS protocol detailed but generalizability limited to expert setting.
Zhang, 2020 [41]	Unclear	Unclear	Low	Low	Prospective design; unclear patient selection and blinding of index test; surgery + histology as reference standard; low risk overall	Low	Low	Low	Representative surgical population; single expert examiner; TVS protocol detailed; reference standard appropriate

Supplementary Table S2. QUADAS-2 risk of bias and applicability assessments with per-study justifications.

No.	First author and year	Site(s) assessed	Patient preparation	Adjunctive techniques	Diagnostic framework
1.	Aas-Eng, 2020 [13]	Rectosigmoid	None	No gel, saline, or rectal contrast instillation	Measurement method based on lesion-to-anal-verge distance (LAVD):<br>– *Method 1* – For rectovaginal septum lesions, manual measurement of probe length from anal verge.<br>– *Method 2* – For higher lesions, composite measurement adding two distances (lesion–cervix + cervix–anal verge).
2.	Aas-Eng, 2021 [14]	Rectosigmoid	None	No gel, saline, or rectal contrast instillation	Measurements obtained for three lesion dimensions — *length, thickness, transverse diameter*<br>Mean TVUS vs. pathology: 43.2 × 19.9 × 10.8 mm;<br>ICC = 0.82, 0.76, 0.58.
3.	Aas-Eng, 2023 [15]	Rectosigmoid	None	No gel, saline, or rectal contrast instillation	Measuring methods for lesion-to-anal-verge distance (LAVD):<br>– *Method 1* – Lesions at/below rectovaginal septum: probe tip placed at lesion's caudal end, index finger at anal verge; measured on probe with ruler.<br>– *Method 2* – Lesions above rectovaginal septum: distance from lesion to posterior cervix (frozen image) + distance from cervix to anal verge.
4.	Abrao, 2023 [16]	Rectosigmoid, USL, PDO	Glycerol rectal enema	Combined transvaginal and transabdominal ultrasound	IDEA protocol; lesions staged with AAGL 2021 classification (I–IV) based on ultrasound findings; compared with surgical AAGL stage.
5.	Arion, 2019 [17]	PDO	None	No gel, saline, or rectal contrast instillation	Compared two preoperative tests for prediction of PDO:<br>– Pelvic examination for nodularity in posterior fornix.<br>– TVUS sliding sign performed immediately afterward.<br>Reference standard: laparoscopic confirmation
6.	Asgari, 2022 [18]	Rectosigmoid, USL, PDO	Saline laxative enema<br>Soft diet day before<br>No breakfast on day of scan	Combined transvaginal and transrectal ultrasound	IDEA protocol; deep endometriosis defined as > 5 mm subperitoneal infiltration.
7.	Bailey, 2024 [19]	USL, PDO	None	No gel, saline, or rectal contrast instillation	Measured hypoechoic endometriosis lesions in three orthogonal planes; peritoneal penetration cut-off of 5 mm used to distinguish superficial versus deep endometriosis.
8.	Barra, 2021 [20]	Rectosigmoid, USL	Rectal enema (133 mL monobasic sodium phosphate)	Rectal water contrast: instillation of ~300 mL sterile saline into the rectum under ultrasound control via catheter<br>Sonovaginography: 40 mL ultrasound gel instilled into the posterior vaginal fornix	Definition of nodule morphology per IDEA: hypoechoic thickenings or nodules with irregular margins and hyperechoic foci; assessment of submucosal infiltration, lesion-to-anal-verge distance, and bowel lumen stenosis.

9.	Brătilă, 2016 [21]	Rectosigmoid, USL, PDO	None	Sonovaginography: 40 mL ultrasound gel instilled into the posterior vaginal fornix	Lesion criteria: hypoechoic nodules or thickenings, asymmetric USLs, or irregular margins.
10.	Chen, 2025 [22]	USL	None	30–40 mL sterile saline infused into uterine cavity via contrast catheter; saline flowed through fallopian tubes into the pelvis, filling the Pouch of Douglas	Indicators for USL deep endometriosis: thickened ligament, irregular contour, low echogenicity, well-defined margins, absence of Doppler flow.<br>Mean USL nodule size: 13.3 × 8.1 mm with fluid vs. 10.2 × 6.1 mm without ($p < 0.001$).<br>Fluid improved boundary definition and lesion conspicuity.
11.	Di Giovanni, 2018 [23]	Rectosigmoid	Glycerol rectal enema	Combined transvaginal and transabdominal ultrasound Tenderness-guided	Lesions defined as regular/irregular hypoechogenic masses replacing muscularis; bowel DIE confirmed when infiltration > 5 mm.<br>Rectal vs. sigmoid location classified by distance from anal verge (≤ 12 cm = rectum).<br>All missed sigmoid nodules > 25 cm from anal verge (outside ultrasound field).
12.	Di Giovanni, 2022 [24]	USL	None	Combined transvaginal and transabdominal ultrasound	Deep endometriosis diagnosed on ultrasound when irregular, avascular or poorly vascularized hypoechogenic tissue disrupted normal USL/parametrial architecture.<br>Detection limit: 2 mm lesion size.
13.	Ferrero, 2019 [25]	Rectosigmoid	Low-residue diet<br>Oral laxative the day before<br>Rectal enema (120 mL sodium diphosphate) a few hours before imaging	No gel, saline, or rectal contrast instillation	Lesions defined as hypoechoic thickening or nodules of muscularis propria ± hyperechoic foci.<br>Distance from anal verge estimated by probe retraction to perineal plane.
14.	Freger, 2024 [26]	USL	None	Probe inserted into posterior vaginal fornix, angled toward the rectum, then rotated (≤45°) clockwise or counterclockwise to visualize each USL	Three orthogonal plane measurements obtained for each lesion (length, width, height).<br>Mean lesion size examples:<br>– Left USL: 9.3 × 5.0 × 10.0 mm<br>– Right USL: 10.4 × 6.8 × 8.5 mm
15.	Goncalves, 2021 [27]	Rectosigmoid, PDO	Oral laxative the day before<br>Rectal enema (120 mL sodium diphosphate) one hour before imaging	Combined transvaginal and transabdominal palpation	Partial obliteration: Normal peritoneum visible below uterosacral ligaments.<br>Complete obliteration: No peritoneal tissue visible below these structures.<br>Lesion size thresholds: < 1 cm = stage 1, 1–3 cm = stage 2, > 3 cm = stage 3.
16.	Kamkarfar, 2022 [28]	USL	None	No gel, saline, or rectal contrast instillation	Lesion criteria: irregular hypoechoic nodules, wall thickening, retractable masses, or hypoechoic points

					along uterosacral ligaments (USLs), rectosigmoid, bladder, or ureter.
17.	Leonardi, 2020 [29]	PDO	None	Saline-infusion: instillation of ≥ 30 mL sterile saline into the pouch of Douglas via an intrauterine balloon catheter with 1.5 mL sterile water to seal the cervix	Lesions < 5 mm depth from peritoneum defined as superficial endometriosis.<br>Ultrasound features pre-defined as: hyperechoic projections, hypoechoic areas, filmy adhesions, cystic areas, peritoneal pockets with "entrapped" fluid.
18.	Leonardi, 2022 [11]	Rectosigmoid, USL, PDO	Empty bladder before scan	Combined transvaginal and transabdominal ultrasound (as needed)	Used the IDEA consensus four-step approach:<br>– Systematic evaluation of uterus and adnexa (including endometriomas, adenomyosis).<br>– Assessment of "soft markers" — site-specific tenderness and ovarian mobility.<br>– Evaluation of the pouch of Douglas (POD) via the sliding sign (positive = free movement; negative = obliteration).<br>– Detection and mapping of deep endometriotic nodules in anterior and posterior compartments.
19.	Menakaya, 2016 [30]	Rectosigmoid, USL, PDO	None	Gel sonovaginography: 20 mL sterile ultrasound gel introduced into the posterior fornix	UBESS staging I–III:<br>Stage I: Normal ovaries, no DIE, no PDO ± SST → mild disease.<br>Stage II: Endometrioma ± immobile ovaries ± non-bowel DIE → moderate disease.<br>Stage III: Bowel DIE ± immobile ovaries ± PDO → severe disease.
20.	Maple, 2025 [31]	USL	None	Tenderness-guided transvaginal ultrasound	Diagnostic criteria:<br>– Normal: thin, smooth, linear, hyperechoic, homogeneous USL sliding freely over the vaginal wall.<br>– Abnormal: thickened, heterogeneous or hypoechoic USL with regular/irregular margins or hypoechoic nodules fixed to adjacent structures.<br>USL considered thickened if > 3 mm; > 5 mm indicated more advanced disease.
21.	Padmehr, 2023 [32]	Rectosigmoid, PDO	None	No gel, saline, or rectal contrast instillation	The study emphasized that transvaginal ultrasound can precisely map bowel and cul-de-sac DIE, facilitating surgical planning, but ureteral and tubal lesions remain challenging.
22.	Pattanasri, 2020 [33]	Rectosigmoid, USL, PDO	Reproted in 19% of the patients	No gel, saline, or rectal contrast instillation	DIE diagnosis = lesions > 5 mm deep below peritoneum, confirmed on laparoscopy + histopathology.
23.	Reid, 2018 [34]	Rectosigmoid	None	No gel, saline, or rectal contrast instillation	Defined rectal DIE = lesion between anal sphincter and rectum at uterine fundus level; rectosigmoid DIE = lesion at uterine fundus level.<br>Dynamic "sliding sign" test: negative if anterior

					rectum/rectosigmoid failed to glide over posterior cervix or fundus.
**24.**	Ros, 2017 [35]	Rectosigmoid	Saline laxative enema	No gel, saline, or rectal contrast instillation	Key quantitative data (rectosigmoid nodules):<br>– Distance from anal verge ≈ 10.3–10.6 cm<br>– Mean nodule size ≈ 30 × 9 × 13 mm<br>– Mean bowel circumference involvement: 26.5%
**25.**	Ros, 2021 [36]	USL	3-day low-residue diet + two rectal enemas (250 mL sodium diphosphate)	No gel, saline, or rectal contrast instillation	Definition of DE: Endometriotic infiltration > 5 mm.<br>Mean nodule size: USL = 12.1 ± 4.4 mm
**26.**	Sadighi, 2023 [37]	USL, PDO	None	Combined transvaginal and transabdominal palpation	DIE diagnosis: hypoechoic or isoechoic solid nodules/masses with irregular margins, tenderness on palpation, fixed to surrounding structures.<br>DIE cut-off: > 3 mm.
**27.**	Sloss, 2022 [38]	Rectosigmoid	Saline laxative enema	No gel, saline, or rectal contrast instillation	Lesions were assessed for size, location, depth of wall invasion, percentage of circumference involved, and distance from anal verge.<br>Depth of infiltration categorized as superficial (serosa) or deep (muscularis/submucosa/mucosa) based on histopathology.
**28.**	Venkatesh, 2020 [39]	PDO	None	20 mL sterile ultrasound gel instilled into the posterior fornix via syringe	Non-bowel DIE identified as hypoechoic nodules, plaques, or linear thickenings within anterior, posterior, or lateral compartments.<br>Bowel DIE identified as non-compressible hypoechoic lesions in the muscularis propria of the anterior rectal wall, traced up to ~25–30 cm from the anal verge.
**29.**	Yin, 2020 [40]	Rectosigmoid, USL	Saline laxative enema	Ultrasound transmission gel in the probe cover was used as a stand-off to improve near-field visualization.	Lesion morphologies characterized per site:<br>– USL: thickened root (50.6%), nodular (27.4%), cable-shaped (16.5%).<br>– Intestinal: "Indian headdress" sign (32%), segmental low-echo thickening (68%).
**30.**	Zhang, 2020 [41]	USL	None	Ultrasound transmission gel in the probe cover was used as a stand-off to improve near-field visualization; Tenderness-guided	Lesions classified morphologically into four types based on ultrasound appearance:<br>– Type I: Thickened, stiff lesions (root segment of USL thickened, hypoechoic).<br>– Type II: Local nodules (round or stellate, regular/irregular margins).<br>– Type III: Irregular striped lesions (along USL or into adjacent organs).<br>– Type IV: Mixed lesions (combining previous types, often bilateral).

LAVD = Lesion-to-Anal-Verge Distance; **ICC =** Intraclass Correlation Coefficient; **USL =** Uterosacral Ligaments; **PDO =** Pouch of Douglas Obliteration; **IDEA =** International Deep Endometriosis Analysis group; **AAGL =** American Association of Gynecologic Laparoscopists; **UBESS =** Ultrasound-Based Endometriosis Staging System; **DIE =**

Deep Infiltrating Endometriosis**; SST =** Site-Specific Tenderness

Supplementary Table S3. Summary of transvaginal ultrasound protocols and diagnostic frameworks used across the studies included in this systematic review.
